# Vestibular Findings on the Video Head Impulse Test (vHIT) in Pregnancy: A Cross-Sectional Study

**DOI:** 10.7759/cureus.41059

**Published:** 2023-06-27

**Authors:** Melissa Castillo-Bustamante, Ireri Espinoza, Omarliv Briceño, Johanna M Vanegas, Maria del Mar Tamayo, Jorge Madrigal

**Affiliations:** 1 School of Medicine, Universidad Pontificia Bolivariana, Medellín, COL; 2 Otoneurology, Centro de Vértigo y Mareo, Mexico City, MEX; 3 Epidemiology and Public Health, Medical School, Universidad Pontificia Bolivariana, Medellin, COL; 4 Intensive Care Unit, Clinica del Prado, Medellin, COL

**Keywords:** semicircular canal function, vestibular labyrinth, video head impulse test, vestibular system, pregnancy

## Abstract

Background

Functional and anatomic changes occur during pregnancy. Some of these changes are in the auditory and vestibular systems. However, there is a lack of information about the functional changes to critical structures that contribute to balance and proprioception. This study aims to evaluate the functions and shifts to the semicircular canals throughout gestation.

Methodology

This is a cross-sectional study. A video head impulse test (vHIT) was performed on all healthy pregnant patients with gestational periods ranging from the 20th to 40th weeks who were admitted to a maternal-fetal care unit. Vestibulo-ocular reflex (VOR) gains in the lateral, posterior, and anterior semicircular canals and gains in asymmetry were obtained.

Results

A significant positive relationship was observed in the right (*R* = 0.1064; *P* = 0.0110) and left (*R* = 0.2993; *P* = 0.0001) lateral semicircular canals as gestational weeks increased. Lower gains were seen at the start of the second trimester for the lateral canals. No significant gains were seen in the anterior or posterior canals throughout pregnancies until labor. No significant gains in asymmetry were detected.

Conclusions

Pregnant females may present vestibular changes in the semicircular lateral canals starting from the 20th week of gestation until labor. Increased gains may be associated with volumetric changes probably given by hormonal actions.

## Introduction

Pregnancy involves several anatomic and functional modifications to various organs and systems, including the vestibular system [1]. Osmolar, electrolytic, vascular, and chemical changes are known to produce disruptions within specific structures involved in balance and gait, such as stria vascularis, spiral ligament, and semicircular canals [1,2]. These are generally attributed to the action of estrogen and progesterone starting at the first week of gestation until labor, leading to imbalance, falls, proprioception, and cognition disturbances [2-4].

Estrogens and progesterone are significantly increased during pregnancy starting in the 20th week of gestation until labor, leading to increased electrolytic imbalances, excessive retention of sodium and water, and increased volumetric changes within critical structures for balance, such as semicircular canals, the utricle, and the saccule [5,6]. An objective assessment of the semicircular canals can be made using the video head impulse test (vHIT). This is a quick, reliable, and noninvasive test for assessing the function of the anterior, posterior, and lateral semicircular canals based on the examination of the vestibulo-ocular reflex (VOR) during rapid head thrust movements [7].

This test aims to assess the functions of the six semicircular canals, comparing the velocities of the head and eye (described as gain) and the difference between both ears (described as asymmetry) [8,9]. When the VOR is functional, the gain should be close to 1.0, with the eyes turning at the same amount and speed as the head and, as a result, able to maintain visual fixation [8,9].

To date, there has only been one study performed on pregnant females with hyperemesis gravidarum using vHIT to assess vestibular changes [10]. However, there is a lack of information about the functional shifts in the semicircular canals of pregnant females starting at the 20th week until labor using vHIT. We conducted a cross-sectional study in one maternal-fetal unit using vHIT to obtain a better understanding of the semicircular canal function throughout pregnancy in participants from the 20th week of gestation until labor.

## Materials and methods

This cross-sectional analysis included pregnant women admitted to the Department of Obstetrics and the Maternal-Fetal Unit of the Clinica Universitaria Bolivariana - Universidad Pontificia Bolivariana from January to March 2023. Ethical approval was obtained from the Institutional Review Board (IRB) of the Universidad Pontificia Bolivariana in December 2022, IRB number 12/2022. Participants were informed about the study, and informed consent was obtained.

Pregnant patients over 18 years of age without arterial hypertension, diabetes mellitus type I or II, hypothyroidism, rheumatologic conditions, or soft tissue disorders and hyperemesis gravidarum were included in this study. Patients with previous neurotologic conditions, such as Meniere’s disease, vestibular neuritis, benign paroxysmal positional vertigo, unilateral or bilateral vestibulopathy, vestibular schwannoma, vestibular migraine, or inner ear malformations, were excluded from this study. Patients with facial fractures, ocular disorders zygomatic fractures, cervical fractures, and limitations for clinical head impulse tests, as well as patients under benzodiazepines, anxiolytics, or sleep medications, were also excluded.

Demographic data, including weeks of gestation, number of pregnancies, and previous history of vertigo, were taken.

Video head impulse test

The examination using vHIT was performed by an experimented otolaryngologist fully trained in otoneurology. The vHIT device used for this study was EyeSeeCam (Interacoustics, VOG, Munich, Germany; model 2022) [11]. Patients were placed and seated on a static surface at a fixed distance of 2 m from the back of the chair to one point of fixation on the wall. An interchangeable eyeglass integrated into a camera system to record eye movements was used. The room was well-lit to ensure small pupils in each patient [12]. Patients were instructed to relax their necks and keep their eyes open and fixate on the target during the procedure. The examiner stood behind the patient, holding the head firmly during head impulses. Instructions were repeated during the head thrust to obtain an optimal awareness of the patient. Head impulses comprised fast horizontal rotational head movements (>120°/s) with a low amplitude, unpredictable in timing and direction [13].

EyeSeeCam software calculated VOR gains and asymmetry. The normal VOR gain for vHIT is 1.0 [14]. The cutoff value for abnormal VOR gain is less than 0.8. Gain asymmetry is calculated based on the formula (gain asymmetry = [left side gain − right side gain]/[left side gain + right side gain]) and is expressed as a percentage using standardized historical methods [15].

Patients with significant unilateral or bilateral vestibular function loss moved their eyes away from the target when their head was pushed in the direction of the damaged labyrinth, and a corrective saccade was observed when the head was pushed (covert saccade) or immediately after the head was pushed (overt saccade) [15].

The main outcome measures for vHIT were the mean gain of VOR for each canal - right anterior (RA), right lateral (RL), right posterior (RP), left anterior (LA), left lateral (LL), left posterior (LP) - and the gain asymmetry of left lateral-right lateral (LLRL), left anterior-right posterior (LARP), and right anterior-left posterior (RALP) [15].

Statistical analysis

All statistical analyses were performed using GraphPad Prism 9.0 (San Diego, CA, USA). A logarithmic simple regression was performed to evaluate semicircular canal changes during pregnancy given by the gain and asymmetry in each gestational week.

## Results

Sixty-five patients (130 ears) were tested. The mean (standard deviation [SD]) of age was 27.7 (5.41) years. The mean (SD) of the gestational week was 31.1 (5.4, range 20.6-40.3). Approximately 70% of the patients reported their current gestation as their first one. Only 18% of pregnant patients presented vertigo during their last pregnancy.

The data regarding the VOR gains of lateral, posterior, and anterior canals of patients are shown in Table 1. We used a VOR gain cutoff value of 0.8, and we compared gains versus weeks of gestation. The data regarding the gain asymmetry of semicircular canals are also shown in Table 1. No significant VOR gain asymmetry was detected in the lateral, posterior, or anterior plane. No overt or covert saccades were detected in any of the patients included in this study. Right and left anterior semicircular canals did not show any significant differences throughout pregnancy. No significant gain increase occurred throughout pregnancy until labor was seen in these canals. No significant increase in asymmetry was detected in LARP, RALP, or lateral planes throughout pregnancy (Table 1).

**Table 1:** Gain and asymmetry in pregnant patients. RA, right anterior; RL, right lateral; RP, right posterior; LA, left anterior; LL, left lateral; LP, left posterior; LARP, left anterior-right posterior; RALP, right anterior-left posterior; SD, standard deviation

Canals	Gains, mean (SD)	Range
RA	0.92 (0.16)	0.48-1.55
RL	1.02 (0.26)	0.53-1.7
RP	0.95 (0.22)	0.36-1.45
LA	0.89 (0.17)	0.44 -1.3
LL	1.04 (0.25)	0.54-1.5
LP	0.90 (0.15)	0.4-1.2
LARP asymmetry	9,47 (7.15)	1-40
RALP asymmetry	10.16 (9.05)	1-35
Lateral asymmetry	10.52 (8.45)	1-35

**Figure 1:**
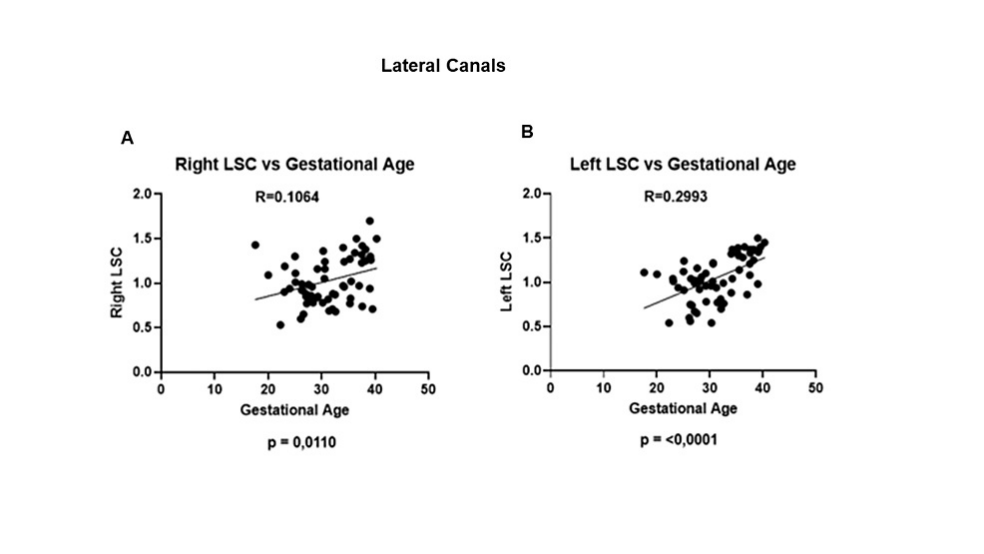
Lateral canals (right and left) gains throughout pregnancy from the 20th week to labor. Significantly increased gains were observed throughout pregnancy starting at the 20th week of gestation. Herein, this progressive increase is seen in the (A) right LSC and (B) left LSC. LSC, lateral semicircular canal

A significant positive relationship was observed in the right (*R* = 0.1064; *P* = 0.0110) and left (*R* = 0.2993; *P* = 0.0001) lateral semicircular canal gains when gestational weeks increased (Figures 1A-1B).

**Figure 2:**
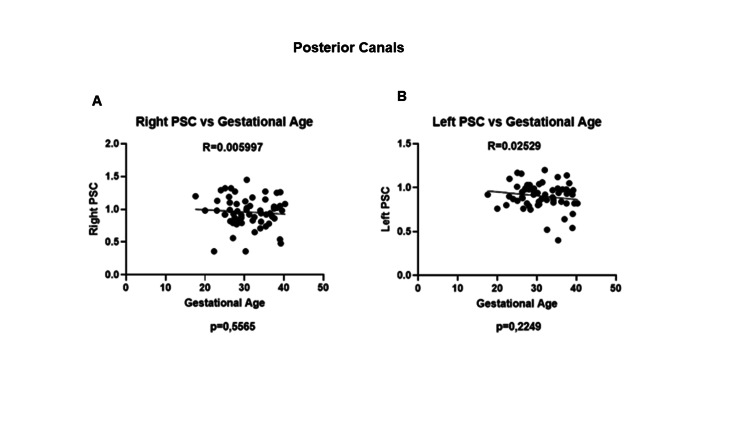
Posterior canals (right and left) gains throughout pregnancy from the 20th week to labor. (A) Right and (B) left PSCs did not show any significant changes in gains throughout pregnancy. PSC, posterior semicircular canal

No differences were reported in the right and left posterior semicircular canals (Figures 2A-2B).

**Figure 3:**
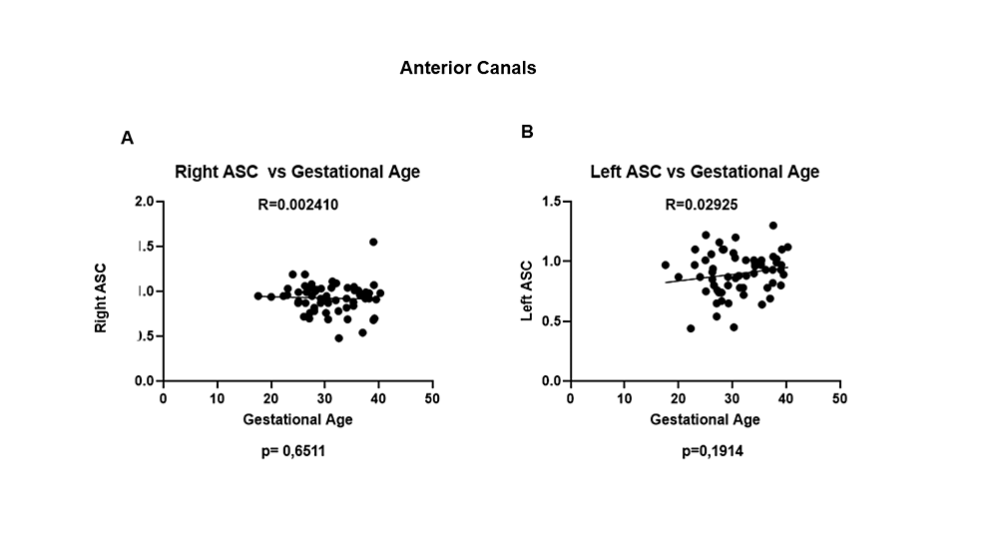
Anterior canals and gains throughout pregnancy. Anterior canals on the (A) right and (B) left presented gains close to the cutoff values reported in the literature. No significant changes were seen throughout pregnancy. ASC, anterior semicircular canal

Right and left anterior semicircular canals did not show any significant differences throughout pregnancy (Figures 3A-3B). 

**Figure 4:**
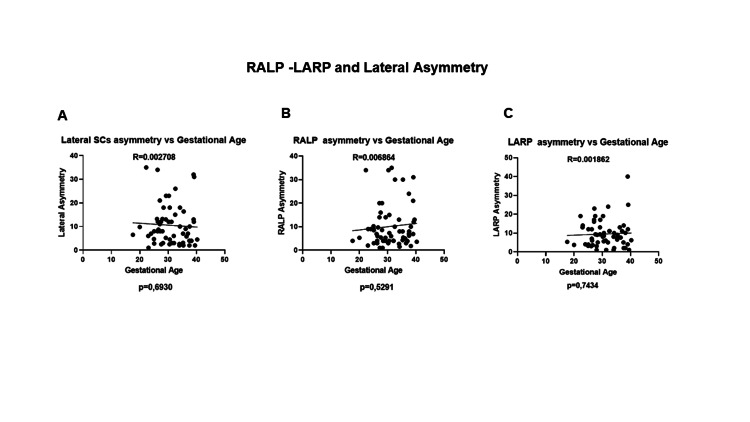
RALP-LARP and lateral asymmetry. No significant differences were seen when asymmetry in the (A) lateral SCs, (B) RALP SCs, and (C) LARP SCs throughout pregnancy. No significant trends were seen in all six SCs. RALP, right anterior-left posterior, LARP, left anterior-right posterior; SC, semicircular canal

No significant increase in asymmetry was detected in LARP, RALP, or lateral planes throughout pregnancy (Figures 4A-4C).

## Discussion

Increased gains and asymmetry were found in pregnant patients close to the beginning of labor starting at the 25th week of gestation. Significantly increased gains were seen in the lateral canals but not in the anterior and posterior semicircular canals. Although this type of gain was found, a few patients referred to vertigo or other vestibular symptoms during pregnancy. To date, this is the first study to reveal these findings in one maternal-fetal unit.

From the first trimester of pregnancy until labor, functional and anatomic changes were reported to the utricle, saccule, and semicircular canals. Vestibular testing, such as cervical vestibular-evoked myogenic potentials (C-VEMP), ocular vestibular-evoked myogenic potentials (O-VEMP), videonystagmography, and vHIT showed decreased amplitudes, decreased gains in posterior and anterior canals, and unilateral weakness and preponderance [10,16]. During the first trimester, reductions in amplitude in C-VEMP and O-VEMP were usually found, indicating a peripheral labyrinthine dysfunction and altered otolithic function [16]. Semicircular canal dysfunction was seen in patients during the second trimester, correlating with low gain values of the left anterior canal and higher asymmetry in the LARP [10]. Saccadic pursuits and persistent positional nystagmus, contralateral preponderance, and increased vestibular unilateral deficits were seen during the first and third trimesters, indicating unilateral dysfunction and altered function of the VOR [10,15]. These findings may be explained by the hormonal influx and electrolytic dysregulation seen during the 20th week of gestation until labor [10,15]. This hypothesis could explain our findings, which revealed increased gains in the lateral semicircular canals. Besides the electrolytic dysregulation and increased endolymphatic influx, increased volumetric changes, the anatomic position of the lateral semicircular canal, and an altered endolymphatic flow may be associated with increased gain [4].

Our study is the first to describe how semicircular canals could be affected by this hormonal influx throughout pregnancy in healthy patients. Formerly, there is only one report in the literature regarding the vHIT evaluation in pregnant patients; however, this was conducted in patients with hyperemesis gravidarum, which is a critical condition that leads to electrolytic disturbances that potentially may affect the optimal functioning of the endolymphatic fluid and the homeostasis in the vestibular system [10]. Vestibular disorders may be unregistered and underdiagnosed. This may represent a challenge for clinicians and healthcare providers as vestibular physical examination and testing are not commonly performed during this time [16]. Besides this, in those patients who are under evaluation, there are some challenges during the procedure that should be considered. One of them is the position of patients during vHIT. Pregnant females may present vascular compression and discomfort during vestibular testing [16]. Also, this population may experiment dizziness and limitation in head movements due to neck pain and cervical changes within the vertebral canal [17,18]. Even though, patients in our study were collaborative and conditions were extremely reviewed and checked, some of them presented discomfort, neck pain, and dizziness during the procedure. Ergonomics and healthcare strategies should be created to avoid this potential risk of bias during vHIT. 

This study presents some limitations. One limitation of the study is the follow-up with patients, which would be critical to get a better understanding of the progressive and prospective changes throughout pregnancy. Further prospective and experimental studies with weekly follow-ups should be made to gain a better understanding of functional changes in the vestibular system during pregnancy and labor.

Another limitation of our study is the lack of information in the literature regarding the vestibular testing used during pregnancy and the vestibular evaluation of patients who presented vestibular disorders during this time. For us, is a challenge that there is no available study to compare normal gains and asymmetry of the semicircular canals in this population, which has strengthened our discussion. 

This study is the first to report increased gains in healthy pregnant females. These findings have not previously been described and are the first to describe expected gain and asymmetry values during this lifetime.

## Conclusions

Pregnant females may present vestibular changes within the semicircular canals, mainly at lateral canals starting from the 20th week of gestation until labor. Increased gains may be associated with volumetric changes probably given by increased endolymphatic flow at the posterior labyrinth. Although there are no other significant changes in the posterior and anterior canals and no semicircular canal asymmetry was described, these are the first changes reported in one representative sample of pregnant patients in the literature. Further prospective and histopathologic studies must be conducted to obtain a better understanding of the phenomena in this population.
